# The impact of decision aids to enhance shared decision making for diabetes (the DAD study): protocol of a cluster randomized trial

**DOI:** 10.1186/1472-6963-12-130

**Published:** 2012-05-28

**Authors:** Annie LeBlanc, Kari L Ruud, Megan E Branda, Kristina Tiedje, Kasey R Boehmer, Laurie J Pencille, Holly Van Houten, Marc Matthews, Nilay D Shah, Carl R May, Barbara P Yawn, Victor M Montori

**Affiliations:** 1 Department of Health Sciences Research, Division of Health Care Policy and Research, Mayo Clinic, Rochester, MN, USA; 2 Knowledge and Evaluation Research Unit, Mayo Clinic, 200 First Street SW, Rochester, MN, 55905, USA; 3 Center for the Science of Healthcare Delivery, Mayo Clinic, Rochester, MN, USA; 4 Department of Health Sciences Research, Division of Biomedical Statistics and Informatics, Mayo Clinic, Rochester, MN, USA; 5 Department of Anthropology and Sociology, Université Lumière Lyon 2, Lyon, France; 6 Department of Research, Olmsted Medical Center, Rochester, MN, USA; 7 Department of Family and Community Health, University of Minnesota, Minneapolis, MN, USA; 8 Family Medicine, Mayo Clinic, Rochester, MN, USA; 9 Faculty of Health Sciences, University of Southampton, Southampton, UK; 10 Department of Medicine, Division of Endocrinology, Mayo Clinic, Rochester, MN, USA

**Keywords:** Diabetes, Shared decision making, Cardiovascular prevention, Implementation

## Abstract

**Background:**

Shared decision making contributes to high quality healthcare by promoting a patient-centered approach. Patient involvement in selecting the components of a diabetes medication program that best match the patient’s values and preferences may also enhance medication adherence and improve outcomes. Decision aids are tools designed to involve patients in shared decision making, but their adoption in practice has been limited. In this study, we propose to obtain a preliminary estimate of the impact of patient decision aids vs. usual care on measures of patient involvement in decision making, diabetes care processes, medication adherence, glycemic and cardiovascular risk factor control, and resource utilization. In addition, we propose to identify, describe, and explain factors that promote or inhibit the routine embedding of decision aids in practice.

**Methods/Design:**

We will be conducting a mixed-methods study comprised of a cluster-randomized, practical, multicentered trial enrolling clinicians and their patients (n = 240) with type 2 diabetes from rural and suburban primary care practices (n = 8), with an embedded qualitative study to examine factors that influence the incorporation of decision aids into routine practice. The intervention will consist of the use of a decision aid (*Statin Choice* and *Aspirin Choice,* or *Diabetes Medication Choice*) during the clinical encounter. The qualitative study will include analysis of video recordings of clinical encounters and in-depth, semi-structured interviews with participating patients, clinicians, and clinic support staff, in both trial arms.

**Discussion:**

Upon completion of this trial, we will have new knowledge about the effectiveness of diabetes decision aids in these practices. We will also better understand the factors that promote or inhibit the successful implementation and normalization of medication choice decision aids in the care of chronic patients in primary care practices.

**Trial registration:**

NCT00388050

## Background

Type 2 diabetes mellitus is a metabolic derangement of epidemic impact that lowers the quality and duration of life for millions [1]. The public health impact of this epidemic is best understood when considering that diabetes is associated with an increased risk of premature death (mostly from cardiovascular causes), cardiovascular disease, blindness, renal failure, chronic neuropathic pain, and limb amputations. Successful management of type 2 diabetes requires preventive care and incorporation of healthy nutritional and activity habits. Pharmacologic therapy to achieve metabolic control and favorably impact risk factors associated with diabetes-related complications is almost always necessary. Several medications to control hyperglycemia, hypertension, and dyslipidemia have become available in the last decades. Patients who experience the use of these medicines, often in combination, and their costs and side effects often report their burden to exceed the perceived burden of diabetes complications [2,3].

A patient-centered approach to care that promotes patient involvement in selecting the intensity and components of a complex diabetes medication program to better match patient values and preferences contributes to high quality care and may enhance medication adherence [4]. Decision aids, tools to help involve patients in decision making by clearly and accessibly presenting the available options and their relative advantages and disadvantages, may facilitate patient-centered care [5]. In close collaboration with a multidisciplinary team of patients, clinicians, and designers, we have developed decision aids targeting glycemic and cardiovascular risk factor control through medication therapy (*Diabetes Medication Choice, Statin Choice*) [6,7]. These tools have been shown to improve patient knowledge, involvement in the decision making process, and satisfaction with healthcare [6,8]. In addition, ninety percent of the clinicians considered the decision aids helpful and would use these decision aids in their practice [8].

Despite their efficacy [5], the adoption of decision aids in practice has been dismal for reasons that remain unclear. Furthermore, limited evidence supports our understanding of how decision aids become routinely implemented, embedded, and sustained in practice—that is, how they become normalized. Current approaches to understanding the implementation of decision aids in practice have included analyses of the barriers to using decision aids [9], and problems of individual behavioral change [10] or organizational diffusion. To our knowledge, there is no practice-based research focused on how to normalize these decision aids in the routine of busy clinical practices and on evaluating the effectiveness of diabetes decision aids on patient, clinician, and practice outcomes. Thus, there is urgent need to conduct patient-centered translational practice-based research in diabetes care.

To pursue this effort we propose to 1) evaluate, in a cluster-randomized practical trial enrolling primary care practices and their patients with type 2 diabetes, the impact of patient decision aids vs. usual care on measures of patient involvement in decision making, diabetes care processes, medication adherence, glycemic and cardiovascular risk factor control, and resource utilization; and 2) identify, describe, and explain, using a theory-driven qualitative research approach, factors that promote or inhibit the routine embedding of decision aids in the rural and suburban practices participating in the randomized trial.

## Methods

### Study design

**Figure 1:**
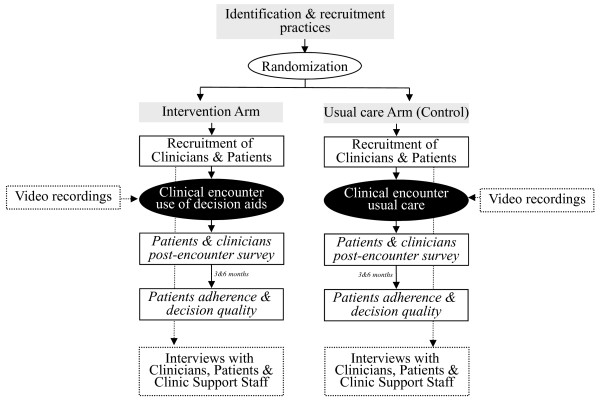
Study design.

We will be conducting a mixed-methods study comprised of a cluster-randomized, practical, multi-centered trial enrolling primary care practices with a qualitative study embedded to examine how the practices will be incorporating the decision aids into their clinical routines (Figure 1). The Mayo Clinic and Olmsted Medical Center Institutional Review Boards (IRB) have approved the study procedures described herein.

### Setting

We will recruit clinicians and their patients with type 2 diabetes receiving routine diabetes care from participating primary care practices of Olmsted Medical Center across rural and suburban communities in Southeastern Minnesota, USA.

### Study participants and eligibility criteria

**Figure 2:**
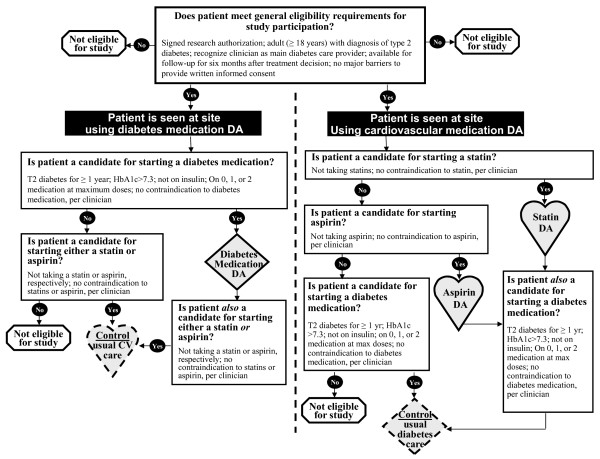
Eligibility criteria and assignment by arms.

***Clinical practices.*** Eligible practices are those that have prioritized chronic disease care as an area for quality improvement and have served at least 50 patients with type 2 diabetes in the 12 months prior to the eligibility assessment. ***Primary care clinicians.*** We will recruit clinicians, defined as professionals with patient healthcare responsibilities (i.e., physicians, nurse practitioners, physician assistants), from participating practices if they are providing care to adults with type 2 diabetes. ***Patients.*** We will consider eligible adult patients (≥18 years) with type 2 diabetes if they recognize the participating clinician as their main diabetes care provider, have no major barriers to provide written informed consent (e.g., severe hearing impairment, dementia), can communicate in English, and declare being available for a six-month follow-up. Furthermore, these patients should need to start, intensify, or modify their antihyperglycemic treatment. Additional criteria will be used to identify eligibility according to designated study arms (Figure 2).

### Participant recruitment

We will send an introductory letter providing an overview of the study and inquiring about potential interest to eligible practices. Study team members will then personally visit interested clinics to discuss the study in more detail with clinic leaders. We will identify a lead clinician (“clinical champion”) to become the contact person for each practice for the duration of the study and seek participation of clinicians during this first meeting when the study is discussed. Potentially eligible patients will be identified through the diabetes registry, and we will note their upcoming appointments with participating clinicians. A study team member will contact these patients in advance to seek their participation in the study. Trained study personnel will obtain written informed consent to participate in the study from both clinicians and patients prior to their clinical encounter.

### Allocation procedures

Randomization will be by practice. The study will compare the use of the decision aids within the clinical encounter versus usual care. We will identify pairs of practices that are most similar in size (i.e., number of clinicians seeing adult patients with type 2 diabetes) and randomize within each pair to use either the 1) the *Statin Choice* and the *Aspirin Choice*, or 2) the *Diabetes Medication Choice* decision aid. Each practice will also serve as the usual care arm for the other intervention arm (Figure 2). This approach will seek to ensure similar representation of ‘easy’ and ‘difficult’ practices in both decision aid groups and eliminate the need for any site to simply serve as a usual care arm. A study statistician will perform the randomization centrally after the practices have been enrolled, ensuring concealment of allocation of the paired practices. Practices, clinicians, patients, and investigators will not be masked to the intervention. However, patients providing outcomes will remain masked to their practice status, as consent documents will keep patients unaware of the study’s main hypotheses. Furthermore, we will centrally follow patients and ensure that patient surveys and pharmacy follow-up are completed according to the intention-to-treat principle.

### Intervention

The intervention will consist of the use of a decision aid (*Statin Choice* and *Aspirin Choice,* or *Diabetes Medication Choice*) by patients and their primary care clinician during the clinical encounter (http://kercards.e-bm.info).

#### Decision aids

The *Statin Choice* decision aid has three different versions according to baseline 10-year cardiovascular risk, pre-assessed for each patient: 10% (used for patients with a 10-year cardiovascular risk <15%), 20% (for patients with estimated risk between 15 and 30%), and 50% (for patients with estimated risk >30%) [6,11]. The decision aid also presents the absolute risk reduction of cardiovascular events with statins, the potential downsides of taking statins, and a question prompting patients to express whether they are ready or not to make a decision and consequently which action they would like to take. The *Aspirin Choice* decision aid is similar to the *Statin Choice* decision aid with the only difference being that it presents the absolute risk reduction of cardiovascular events with aspirin. The *Diabetes Medication Choice* decision aid takes the form of cards that compare commonly used diabetes medication classes across several domains (e.g., reduction in HbA1c, weight gain, cost, mode of administration) that patients with type 2 diabetes and clinicians consider important when choosing these medications [7,8].

#### Training of clinicians

A study team member will conduct a demonstration showing how to use the decision aid at the time of the initial in-person discussion with clinics. The focal points of the demonstration will be that decision aids serve as guides for conversation rather than scripted discussions; that clinicians have flexibility in the manner in which they use the decision aid, including how and when they use them during the visit; and that they may elect not to use the tools with certain enrolled patients, per their own judgment. Brief video clips and storyboards that demonstrate the basic use of decision aids are publicly available at http://kercards.e-bm.info for clinicians to review at their convenience. A study team member will remain available to do one-on-one demonstrations after the initial group demonstration if needed.

### Usual care

For patients in the usual care arm, clinicians will manage the discussion about medication regimen as usual, without using decision aids.

### Frameworks

The RE-AIM framework will guide this study (aim 1). This framework has been developed specifically to address how an intervention, in this case use of decision aids, is implemented in a real-world setting [12,13]. Dimensions of the RE-AIM include: *Reach* (how broadly is this intervention used within the practices), *Effectiveness* (what is the impact of the intervention on outcomes), *Adoption* (can this be adopted by new groups with ease and minimal modifications), *Implementation* (what are the special issues and barriers in implementation), and *Maintenance* (can the intervention be maintained and will the impact continue) [12,13]. To complement the RE-AIM framework, we will use the Normalization Process Theory (aim 2) [6,14]. This applied theoretical model focuses attention on the practical work that facilitates understanding of the factors that promote and inhibit the routine embedding of interventions, such as decision aids into practice, in a structured and parsimonious way. The combination of an established implementation framework and robust explanatory model will provide a strong foundation for process evaluation of the trial.

### Data collection & analysis

#### Aim 1. Evaluating the impact of patient decision aids

We will collect patients’ data through 1) self-reported questionnaires administered before and after the clinical encounter with their clinician, and at 3 and 6 months post encounter; and 2) information about diabetes-related care included in medical records. Participating clinicians will be given a brief questionnaire to complete immediately following each clinical encounter with a participating patient. In addition, we will videotape each clinical encounter. Practice data will be obtained from administrative and patient medical records.

##### Outcomes measures

To assess *Effectiveness* of the decision aids (R**E**-AIM) we will measure the decisional conflict as the primary outcome and patient involvement in decision making, diabetes care processes, medication adherence, glycemic and cardiovascular risk factor control, and resource utilization as secondary outcomes, in the following way:

· *Patients’ decisional conflict*. Patients will complete, immediately after the clinical encounter with their clinician, a modified version of the Decisional Conflict Scale [15], the most commonly used outcome measure in decision aid trials [5]. These modifications entail the use of brief items that explore the quality of the deliberation process during the visit. Psychometric properties include good internal consistency, test–retest reliability, and effect sizes for responsiveness to change ranging from 0.4 to 1.2 [15,16].

· *Patients’ involvement in decision making*. We will use the OPTION scale to assess the extent to which clinicians seek to engage patients in decision making [17,18]. OPTION is a third-person observer scale and was designed for use in reviewing audio recordings of primary care visits. Our group has extended the use of this tool to video recordings with excellent inter-rater reliability (intraclass correlation coefficient >0.7) [6,19,20].

· *Patients’ knowledge*. Patients will complete a knowledge questionnaire immediately following the encounter with their clinicians, and at 3 and 6 months post-encounter. The questionnaire was developed according to prior recommendations and is similar to questionnaires used in our previous studies, addressing general knowledge about diabetes and lipid management and specific information contained in the decision aids [6,8].

· *Patients’ satisfaction*. Satisfaction with decision making will be assessed using items from the Decisional Conflict Scale [15] as well as two specific questions that require patients to assess the extent to which they would want for themselves and recommend to others similar decision support as they received during the visit. Other questionnaires exist to specifically address this domain, but our use of the Decisional Conflict Scale for this purpose reflects our effort to minimize participant burden.

· *Patients’ quality of life*. Patients will assess their health-related quality of life prior to the clinical encounter through the use of the EuroQol EQ-5D scale in which they list their health as excellent, very good, good, fair or poor [21]. The EQ-5D has been used in primary care [22,23] and in diabetes care and diabetes trials [24,25].

· *Patients’ hemoglobin A1c, lipids, blood pressure, & body mass index.* Medical records will be the source of information about these commonly used intermediate outcome measures for diabetes [26,27].

· *Patients’ prescription drugs.* Prescription drug and billing data will be collected through pharmacy records. Patients will be asked to provide written authorization to contact their pharmacy and release this information to the investigators. We have been able to obtain complete pharmacy records for all of our participants in previous trials [6,8].

· *Patients’ adherence*. Adherence and persistence measures will be derived from patient self-report, metabolic outcomes (indirect measure), and pharmacy records. These diverse sources are necessary given the nature of medications (e.g., multiple dosing and dose adjustments, particularly of insulin, for diabetes medication) [28,29].

· *Clinicians’ satisfaction.* Clinicians will complete a brief questionnaire after each encounter where we will ask about their satisfaction regarding the discussion they had with their patient about starting a medication.

· *Costs and resource utilization.* We will collect cost and utilization data from patients using two methods: first, by attempting to collect billing data from all clinicians the patient reports seeing in the six-month period and second, by collecting data on resource utilization from the patient questionnaire at six months. Data collected will include the number of hospitalizations, reason for hospitalization, whether the hospitalization was for medical or surgical condition, and the length of hospital stay. Additionally, we will collect data on emergency room and ambulatory care utilization. This utilization data will be costed according to the methods described by Glick et al. [30]. We will evaluate the economic impact of decision aid implementation from the clinician perspective based on the length of visits. The incremental time for decision aid visits will be considered the opportunity cost of using a decision aid. We will convert this incremental time to potential number of additional diabetes-related visits that could have been scheduled in 12-months in place of decision aid assisted visits. The visits will be costed using the Medicare Fee schedule for a diabetes-related visit. We will add the costs of materials (decision aids) to estimate the total practice-related costs.

To assess the *Reach* of the decision aids (**R**E-AIM), we will use a tracking log to record patients who are enrolled as well as those who declined the invitation to participate. We will use this data to evaluate reach, e.g., the ratio of enrolled patients to invited patients in each clinic and the characteristics of eligible patients who enrolled and declined. This will allow us to measure participation and representativeness.

To assess the *Adoption* of the decision aids (RE-**A**IM), we will estimate the proportion of practices or clinicians who adopt the intervention. Using clinician surveys and medical record review, we will compare the adoption rates across the intervention practices. We will select a random sample of medical records from each site to determine the extent to which the use of the decision aid is discernible in the records, and the extent of use and success when mentioned.

To assess the *Implementation* of the decision aids (RE-A**I**M), we will seek to determine the extent to which the intervention is implemented as intended. Using the video recordings of the clinical encounters, we will assess the fidelity with which the decision aids are delivered and used as intended during these clinical encounters. We have developed a fidelity checklist for each of the decision aids. These checklists have 10 to 12 items and are completed by a third observer reviewing the video recordings of the encounters [20].

To assess the *Maintenance* of the decision aids (RE-AI**M**), we will conduct a site visit and medical record review of eligible patients, approximately six months after removing investigator implementation support from the sites. This medical record review is in addition to the reviews that will take place at three and six months following the study visit.

##### Socio-demographic characteristics

We will ask patients to report demographic information that is not available in the medical record, such as marital status, years of education, occupation, and household income. We will collect the following characteristics for clinicians: type of practice, years in practice and at practice site, gender, birth year, ethnicity, race, estimate of time in direct patient care, proportion of practice devoted to patients with diabetes, and average length of appointments with diabetes patients. Data will be collected to characterize participating practices including race, ethnicity, and insurance status of patients seen in the practice; practice type, community size, and make-up of staff including clinicians, allied health staff, and patient educators.

##### Sample size

A total of 8 practices will be randomized to 1) the Diabetes Medication Choice decision aid plus statin and aspirin usual care or 2) the Statin Choice and Aspirin Choice decision aids plus diabetes medication usual care. The Statin Choice cluster randomized trial evaluated decision quality comparing the decision aid to usual care. This study reported a 9.8 point difference in decision quality^3^ with the standard deviation of 16.9 and 14.1 for the usual care and decision aid groups, respectively. Making the following assumptions: 1) variances are as reported in this study; 2) we seek to detect a difference of 9.8 points or greater in decision quality between two groups at significance level of 0.05, with a two-sided *t*-test; 3) a modest correlation of outcomes across these clinicians and practices (which is a conservative assumption) represented by an intracluster correlation coefficient (ICC = between cluster variance/total variance) of 0.05; 4) a variance inflation or design effect factor [1+ (n − 1) · ICC], where n is the number of patients per cluster [31]; and 5) an approximated 20% attrition rate, we will have 80% power if we are able to recruit 30 patients per clinic for a total recruitment target of 240 patients. Assuming a similar ICC and attrition rate for other outcomes, this sample size will have 99% power to detect a 1 SD difference in any continuous measure (e.g., approximately a 2-point difference in a 10-question knowledge scale), and 80% power to detect a 30% difference in 6-month adherence rates assuming a control adherence rate of 50%. Actual power will be likely greater because we will adjust for baseline values and characteristics and because of our conservative assumptions.

##### Analysis

We will have a summary of the cluster level and patient level characteristics within each trial arm, providing counts and frequencies for categorical variables and means with ranges for continuous variables. Because of the uncertainty as to the ability of a cluster trial to create uniform prognostic groups, we will test the null hypothesis of no difference between arms in baseline cluster level characteristics using the weighted paired *t*-test [32]. This test can account for the small number of clusters and an unequal number of patients within each cluster.

To account for the modified study design (e.g., each arm being both intervention and usual care), the study team assumes similar adherence rates within statin medications and diabetes medications along with similar knowledge, decisional conflict, and satisfaction rates for patients discussing either medication for those within the usual care group. For the decision aids (Statin, Aspirin and Diabetes Medication) the study team assumes the same impact on all of these outcomes for all decision aids. With these assumptions, all patients that use a decision aid during the encounter of interest will be grouped in the decision aid arm and those that do not will be grouped under the usual care arm. To account for differences that may exist between the diabetes, statin, and aspirin patients, all models will be adjusted by group. Interaction between arm and group will be tested for significance, recognizing that the study will be underpowered to test for this interaction.

To evaluate the efficacy of the intervention, differences in all patient level outcomes, for both continuous and dichotomous outcomes (as well as patient characteristics at baseline) will be estimated using the random effects meta-analysis method [31]. This approach has been validated for matched pair cluster-based studies: differences in means for continuous outcomes and in proportions for dichotomous outcomes are estimated and then pooled across strata. A stratum in terms of a matched-pair study is considered to be each pair, and in this trial there are 5 strata. For each outcome, we will thus estimate the effect size and its precision (e.g., 95% confidence interval). The outcomes will be assessed at baseline, 3 and 6 months. We will estimate the intraclass correlation coefficient for each outcome along with its 95% confidence interval and report these for informational purposes only since the number of clusters is insufficient to determine with confidence a true intraclass correlation^124^. Within patients that had a diabetes medication discussion, HbA1c will be collected and categorized to <7.3 versus ≥7.3. This outcome will be analyzed as described above for categorical outcomes. All analysis and data management will be conducted utilizing SAS (version 9, Cary, NC) with use of Stata statistical software (version 11.0, College Station, Tx) for primary endpoint analysis.

Patients with missing outcome data will not be included in assessment of that particular outcome. We will compare the incidence of missing data for each outcome between study arms, both absolutely and as a function of follow-up period. We will report rates of missing data for each outcome by study arm and known reasons for missing data. In the event that the rate of missing data is not independent of study arm or is greater than the assumed 10% loss to follow-up, we will conduct sensitivity analyses under a range of assumptions about the missing values and assess consistency of results across those scenarios. In the event that patients are missing baseline characteristics data, which are otherwise imbalanced between the two study groups, we will use imputations to estimate models which include those characteristics.

#### Aim 2. Identify, describe, and explain factors that promote or inhibit the routine embedding of decision aids in urban and nonurban practices

To evaluate how the decision aids are routinized into primary care practice, we will conduct a qualitative study with a subset of participants (patients, clinicians, and clinic support staff) at the study sites. The qualitative study will have two major components, which will include 1) a qualitative analysis of video-recordings of clinicians/patients clinical encounters in both intervention and usual care arms, and 2) in-depth, semi-structured interviews with participating patients, clinicians, and clinic support staff, in both intervention and usual care arms.

In addition to the video recordings of the clinical encounters, we will conduct semi-structured, in-depth, interviews with participating patients, clinicians, and clinic support staff (Figure1). Patients will be interviewed within a few weeks after their clinical encounter so as to remember the details of the visit. Clinicians will be interviewed after all patient recruitment has been completed so as to reflect on their management of several different patients during this study, both in intervention and usual care arms. Clinic support staff (e.g., receptionists, nurses, and medical assistants) will be interviewed based on their involvement with the study. An experienced qualitative researcher will lead these interviews and audio-record them with participant consent. Interviews will be no longer than one hour and will be held at the participating site, at a time deemed most convenient for the participant. When appropriate, clinicians will review excerpts of their own video-recordings of clinical encounters.

##### Sample size

From the video recordings of clinical encounters from both arms, we will seek to identify a maximum variation sample of 30 encounters, according to patient age (<65 or ≥65), whether the patient is accompanied during the visit, and whether there is gender congruence between patient and clinician. We will conduct between 20 and 40 interviews for both, participating patients and clinicians, in the intervention and usual care arms of the trial. We will conduct between 10 and 20 interviews with clinic support staff, with a purposive sample that are most likely to be affected by the use of the decision aid.

##### Analysis

Patient and clinician interviews and video-recordings of clinical encounters will be fully transcribed, including observational notes of how the decision aids are used. A qualitative analysis team of at least 6 people will conduct data analysis by using standard qualitative content analysis techniques [33-35]. Trustworthiness of the analysis will be ensured by: 1) assessing transcripts for consistency, 2) group coding, 3) coding the first round individually, and 4) coding subsequent rounds reiteratively. The analysis team will identify key themes and will code interview and clinical encounter transcripts in an iterative process. The team will use qualitative data management software (NVivo 9.0) to facilitate data organization and coding. Each transcript will be independently coded by at least two team members to establish internal validity. Consensus will be reached through discussion. Codes, which have quotations assigned to them, will be examined by all analysis team members to ensure consistent code usage. Coded data will be compiled and analyzed in the form of memos as a data reduction strategy to build a conceptual model that resonates with the constructs of Normalization Process Theory [6,14]. An observational guide will be used to analyze the videotaped encounters. The guide is designed to document initiation of discussion, length of discussion, engagement of patient in discussion, topics discussed, use of context, and integration of decision aids into the medication choice discussion.

## Discussion

The proposed trial seeks to determine the impact of patient decision aids vs. usual care on measures of patient involvement in decision making, diabetes care processes, medication adherence, glycemic and cardiovascular risk factor control, and resource utilization in urban and rural practices in the Midwestern United States. Upon completion of this trial, we will have new knowledge about the effectiveness of diabetes decision aids in these practices and about the processes that promote or inhibit the successful implementation and normalization of medication choice decision aids in rural and urban primary care practices.

## Competing interests

The authors of this protocol disclose no financial conflict of interest pertinent to this study. The Knowledge and Evaluation Research Unit at Mayo Clinic houses the processes of design and evaluation of decision aids, such as *Diabetes Medication Choice* and *Statin Choice*, decides on topics of investigation, pursues funding, designs and conducts evaluation trials, and reports their findings. Investigators at the KER unit, including authors of this manuscript, do not receive funding from any for-profit pharmaceutical or device manufacturer, nor do they receive any royalties or other monetary benefits, directly or indirectly, from the use of the decision aids. The KER unit makes effective decision aids available online free of charge at http://kerunit.e-bm.org, http://kercards.e-bm.info/ and http://shareddecisions.mayoclinic.org/.

## Authors’ contributions

AL wrote the first draft of the manuscript. KLR contributed to the first draft of the manuscript and made critical revisions to the manuscript. NDS, BPY, CRM and VMM conceived and designed the study, applied for funding, and made critical revisions to the manuscript. MEB, KT, LJP, and HVH contributed to the design of the study and provided revisions of the manuscript. KRB and MM made critical revisions to the manuscript. All authors approved the final version of this manuscript.

## Pre-publication history

The pre-publication history for this paper can be accessed here:

http://www.biomedcentral.com/1472-6963/12/130/prepub
